# Objective and Subjective Prevalence of Obstructive Sleep Apnoea/Hypopnoea Syndrome in UK Adults with Down Syndrome: A Strong Marker for Diurnal Behavioural Disturbances

**DOI:** 10.3390/brainsci11091160

**Published:** 2021-08-31

**Authors:** Elizabeth A. Hill, Linda J. Williams, Sally-Ann Cooper, Renata L. Riha

**Affiliations:** 1 Sleep Research Unit, Centre for Clinical Brain Sciences, The University of Edinburgh, Edinburgh EH16 4SB, UK; lizzie.hill@ndcn.ox.ac.uk; 2 Edinburgh Clinical Trials Unit, The Usher Institute, The University of Edinburgh, Edinburgh EH16 4UX, UK; linda.williams@ed.ac.uk; 3 Institute of Health and Wellbeing, University of Glasgow, 1st Floor Administrative Building, Gartnavel Royal Hospital, Glasgow G12 0XH, UK; sally-ann.cooper@glasgow.ac.uk; 4 Department of Sleep Medicine, Royal Infirmary of Edinburgh, Edinburgh EH16 4SA, UK

**Keywords:** sleep-related breathing disorders, obstructive sleep apnoea, home sleep apnoea testing, excessive daytime sleepiness, down syndrome, trisomy 21, prevalence

## Abstract

Prior to this study, the prevalence of obstructive sleep apnoea/hypopnoea syndrome (OSAHS) in adults with Down syndrome was unknown. We hypothesized that unrecognised OSAHS could have an additional deleterious impact on mood and behavioural disturbances in this group of people. Aims: To assess the prevalence of OSAHS in adults with Down syndrome in the United Kingdom, subjectively and objectively, and ascertain its association with diurnal behavioural disturbances. Method: Cross-sectional questionnaire study with home sleep apnoea testing (HSAT) during 2011–2015 across the four nations of the United Kingdom. Participants were adults aged ≥16 years with Down syndrome. Main outcome measures were: self- or caregiver-completed questionnaire data, including the Pictorial Epworth Sleepiness Scale (pESS), selected domains of the Developmental Behavioural Checklist for Adults (DBC-A), anthropometric measures, and symptoms of OSAHS. Objective prevalence was undertaken in a sample of responders using HSAT. Results: Responses were received from 1321/5270 participants (25%), with 1105 valid responses (21%). Eighty-one participants (7%) reported a prior diagnosis of OSA, of whom 38 were receiving therapy. Using validated algorithms, a diagnosis of OSAHS was probable in 366 participants (35%), who were younger, with higher BMI and higher mean total pESS (*p* < 0.0001). A total of 23% of participants had a pESS > 10. OSAHS was a strong marker for behavioural disturbances on the DBC-A depression, disruption and anti-social subscales (*p* < 0.001). Of 149 individuals who underwent HSAT, 42% were diagnosed with OSAHS. Conclusions: Untreated OSAHS in Down syndrome is common and associated with behavioural and mood disturbances. Improving awareness of OSAHS amongst adults with Down syndrome, their families and healthcare professionals is essential.

## 1. Introduction

Down syndrome, present in 1 in 1000 live births in Europe [1], is the commonest form of intellectual disability worldwide. Currently, >37,000 people have Down syndrome in England and Wales alone [2]. Life expectancy in people with DS is rising, and may exceed 50 years [3].

Sleep-disordered breathing is characterised by repetitive pauses in breathing during sleep, and affects approximately 24% of the general adult population [4]. Obstructive sleep apnoea/hypopnoea syndrome (OSAHS) is diagnosed when nocturnal apnoea results in significant diurnal symptoms, including excessive daytime somnolence, impaired cognitive function, reduced quality of life, and behavioural and emotional disturbances [4]. The adult prevalence of OSAHS is 2% in women and 4% in men [4]. OSAHS is an independent risk factor for cardiovascular morbidity and mortality, including hypertension, myocardial infarction and stroke [4].

The Down syndrome phenotype includes a flattened face, short neck, generalised hypotonia, loose ligaments, and a tendency towards weight gain—all risk factors for OSAHS. The additional impact of OSAHS on cognitive ability in DS is still unknown, but may have additional deleterious effects [5].

OSAHS prevalence in children with Down syndrome has been reported to be approximately 45–55% [6]; the prevalence in adults with Down syndrome was unknown. Two small studies in adults with Down syndrome (*n* = 6; *n* = 16) reported >80% of their respective samples to have obstructive sleep apnoea, but these studies may not be representative of the wider Down syndrome population [7,8]. A questionnaire study in Canada [9] reported a diagnosis of OSA in 21% of 223 individuals with Down syndrome aged 1 month to >40 years, but prevalence data for adults only were not reported.

The aim of this study was to establish the prevalence and severity of symptoms of OSAHS and related behavioural and emotional disturbances in adults with Down syndrome using subjective and objective measures. We hypothesised that OSAHS would be a strong marker for diurnal behavioural disturbances in adults with Down syndrome.

## 2. Methods

### 2.1. Study Design and Data

The study was approved by the Scotland A Research Ethics Committee (REC no: 11/MRE00/3). Return of a completed questionnaire was considered implicit consent to participate. Individuals undertaking home sleep apnoea testing gave written informed consent.

### 2.2. Subjective Prevalence

Questionnaires and pre-paid reply envelopes were sent to 5266 UK-based adults (≥16 years) with Down syndrome and their caregivers between 2011 and 2014. Potential study participants were identified by local and national organisations supporting people with Down syndrome (see Acknowledgements). 

The questionnaire comprised a section for completion by the individual with Down syndrome and a section for completion by a relative/caregiver. Anthropometric, comorbidity, medication, demographic, and sleep disturbance data (including frequency per week of snoring, witnessed apnoeas, nocturnal choking episodes, frequent awakenings, unrefreshing sleep, and daytime sleepiness) were collected. The pictorial version of the Epworth Sleepiness Scale (pESS) [10], designed to enhance understanding and accessibility in a broader adult population, was also administered. Prior to this study, the pESS had not been used in a population with Down syndrome. 

Additionally, caregivers completed three subscales of the Developmental Behaviour Checklist for Adults (DBC-A) [11], which may be related to sleep deprivation: Disruptive, Anxiety/Antisocial, and Depressive. Subscales were scored as described by Taffe et al. [12]. The DBC-A has been used extensively in populations with people with Down syndrome. 

Medications were classified using the World Health Organisation Anatomical Therapeutic Chemical Classification System [13].

Reported symptoms suggestive of OSAHS were defined using three previously validated algorithms: [14]

1. Snoring ≥ 3 nights/week plus (witnessed apnoeas, or pESS > 10).
2. Snoring ≥ 3 nights/week plus (witnessed apnoeas, or unrefreshing sleep ≥ 3 nights/week).
3. Snoring ≥ 3 nights/week plus (witnessed apnoeas, or daytime sleepiness ≥ 3/week).

### 2.3. Objective Prevalence

In order to assess objective prevalence of OSAHS by sampling as true a cross-section of the target population as possible, responders were considered eligible if aged ≥16 years with Down syndrome and not currently using CPAP; home sleep apnoea testing was offered regardless of subjective sleepiness, sleep-related symptoms, previous sleep diagnoses or any other variables reported in the questionnaire and first recruitment occurred 10 January 2012.

Home sleep studies were conducted using the Embletta^®^ Gold™ (Embla Systems LLC., Amsterdam, The Netherlands) cardiorespiratory polygraphy device. This is a level III device [15], with the capacity to record multiple channels of physiological data. Home sleep apnoea testing by level III polygraphy is routinely used in clinical practice across the UK. Channels were recorded in broad accordance with AASM guidelines for full PSG [16], as recommended by the AASM guidelines for portable monitoring [15], and included nasal airflow and snoring via nasal pressure cannula, respiratory effort via thoracic and abdominal respiratory inductance plethysmography bands, SpO_2_ via pulse oximetry and body position via an inbuilt position sensor.

All studies were manually validated and scored by one of two experienced Registered Polysomnographic Technologists using standard software (Embla^®^ RemLogic™ Embla Systems LLC., Amsterdam, The Netherlands) in broad accordance with current international guidelines [16]. To ensure consistency of scoring, inter- and intra-rater reliability scoring was conducted in randomly selected subsets of 10% of valid studies.

### 2.4. Statistical Analysis

Standard statistical analyses were undertaken using IBM SPSS Statistics for Windows Version 19.0 (IBM Corp., Armonk, NY, USA). 

All variables were checked for normality. Descriptive statistics were generated to report frequencies of sleep problems and participant characteristics. The chi-square test was used for discrete variables, Student’s *t*-test for continuous variables, and Mann–Whitney U test for non-parametric data, to investigate anthropometric, sleep, and behavioural and emotional associations with probable OSA, and gender differences in sleep and behavioural and emotional characteristics. A conservative cut-off for significance was taken at *p* < 0.001 to account for the effects of multiple testing (with alpha = 0.004 to 0.0025 depending on the number of variables). Though a little stringent, we believed that this would lead to mitigating Type I error further in a sample of this size without having to undertake a Bonferroni or Holm correction on each occasion. Pearson’s and Spearman’s rank correlations were used to examine possible associations between anthropometrics, sleep characteristics and behavioural and emotional disturbances. Both binary logistic regression analysis (for exploring a pESS cut-off score below and above 10/24) and generalised linear modelling were undertaken to explore independent associations of OSAHS with anthropometric characteristics, medication use and behaviour/emotion. All analyses were two-tailed. 

Results are presented as mean ± standard deviation for parametric variables or median with interquartile range (IQR 25 to 75%) for non-parametric data, or as number and percentage.

### 2.5. Role of Funding Bodies and Study Sponsors

The Chief Scientist Office (Edinburgh, UK), Baily Thomas Charitable Trust (Luton, UK) and Fondation Jérôme Lejeune (Paris, France) funded this study. ResMed (UK) Ltd. provided non-financial support via loan of home sleep study equipment. None of these parties were actively involved in the design, analysis or reporting of the study. As study sponsors, Lothian Universities Health Trust and the University of Edinburgh did not play any role in the planning, conducting or analysis of this study. 

### 2.6. Public and Patient Involvement

Adults with Down syndrome reviewed the study materials for accessibility via focus groups organised through Down’s Syndrome Scotland, a national organisation supporting people with Down syndrome and their families, with amendments made in line with feedback received. 

## 3. Results

**Figure 1:**
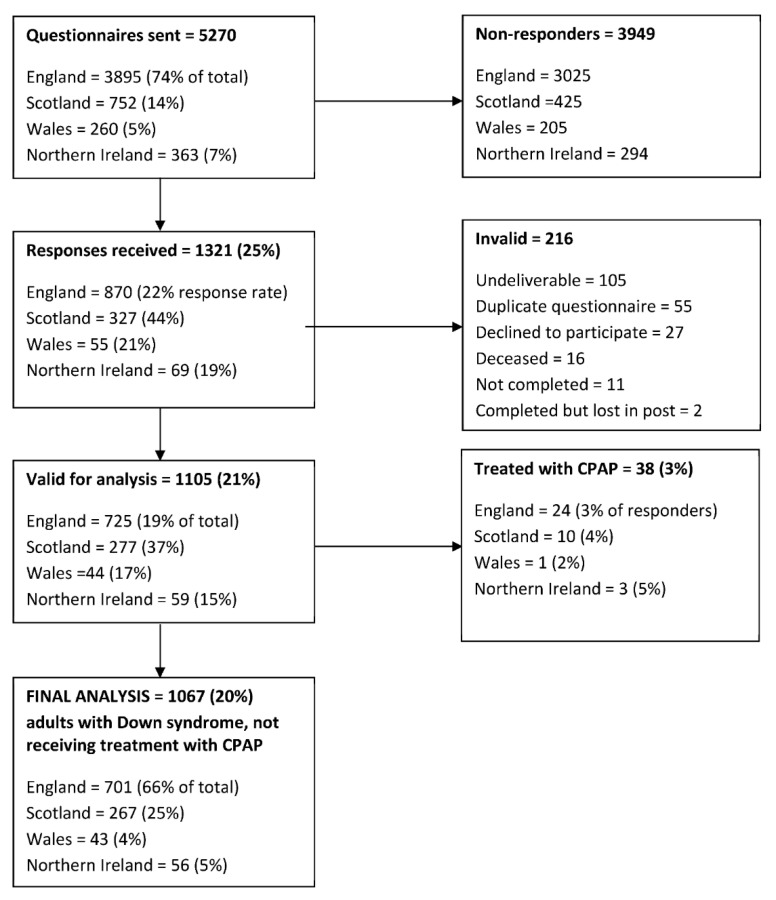
Summary of questionnaires distributed to adults with Down syndrome residing within the UK, returned to the investigators and included in the final analysis.

Responses were received from 1321/5270 participants (25%), of which 1105 responses (21%) were valid for analysis (Figure 1). 

Fifty-three men and 28 women (7%) reported a prior diagnosis of OSA. Of these, 38 (3.4% of the total) were receiving continuous positive airway pressure (CPAP) therapy and were excluded from further analysis. Other reported sleep disorders included insomnia (*n* = 2), narcolepsy (*n* = 1), behavioural sleep problems (*n* = 1), parasomnia (*n* = 1), headbanging (*n* = 1), myoclonic jerks (*n* = 1), nightmares (*n* = 1) and somniloquy (*n* = 1). 

### 3.1. Anthropometric Data and Comorbidities

Anthropometric characteristics of the responders included in the study are shown in Table 1. Most participants were overweight (34%) or obese (40%) [17]. 

Females had a significantly higher body mass index than males (males 28.2 ± 6.6 kg/m^2^, females 30.0 ± 6.8 kg/m^2^; *p* < 0.0001), but smaller collar size (males 41.3 ± 3.8 cm, females 38.2 ± 4.5 cm; *p* < 0.0001). No other significant gender differences were observed.

The prevalence of comorbidities that can be related to OSAHS or potentially affecting sleep (Table 1) was low: hay fever (18%); asthma (13%); epilepsy (6%); diabetes (3%); hypertension (2%); stroke (2%).

Adenotonsillar hypertrophy is known to affect upper airway size and function in both adults and children in the general population. A total of 243 responders (23%) reported removal of tonsils and/or adenoids and 124 (12%) reported adenotonsillectomy. There was a trend for those who had undergone surgery to be more likely to meet the criteria for probable OSAHS (42% vs. 32%, *p* = 0.002; data not shown). 

### 3.2. Sleep Symptoms

Probable OSAHS criteria [16] were met by 366 participants (35%), who were significantly younger, with higher body mass index, higher mean total pESS and more likely to have a pESS > 10 (all *p* < 0.0001) (Table 2 and Table 3 online). Mean pESS scores were within the normal range (7 ± 5), with no significant gender difference (Table 4 online). Excessive daytime somnolence (pESS > 10) was classified in 23% of participants, with no significant gender difference.

The mean self-reported total sleep time was 9.1 ± 1.3 h in 24 h, with 8.6 ± 1.2 h of nocturnal sleep. Most participants (72%) did not take daytime naps. Nocturnal total sleep time was significantly higher in females (<0.0001).

### 3.3. Behavioural and Emotional Disturbances

As hypothesised, OSAHS was a strong marker for diurnal behavioural disturbances; individuals with probable OSAHS scored significantly higher on all three DBC-A domains (Table 2). Independent determinants of sleepiness and behavioural and emotional disturbances are summarised in Table 5 and Table 6, and include use of category N medications, unrefreshing sleep and frequent nocturnal awakenings. 

### 3.4. Home Sleep Apnoea Testing 

**Figure 2:**
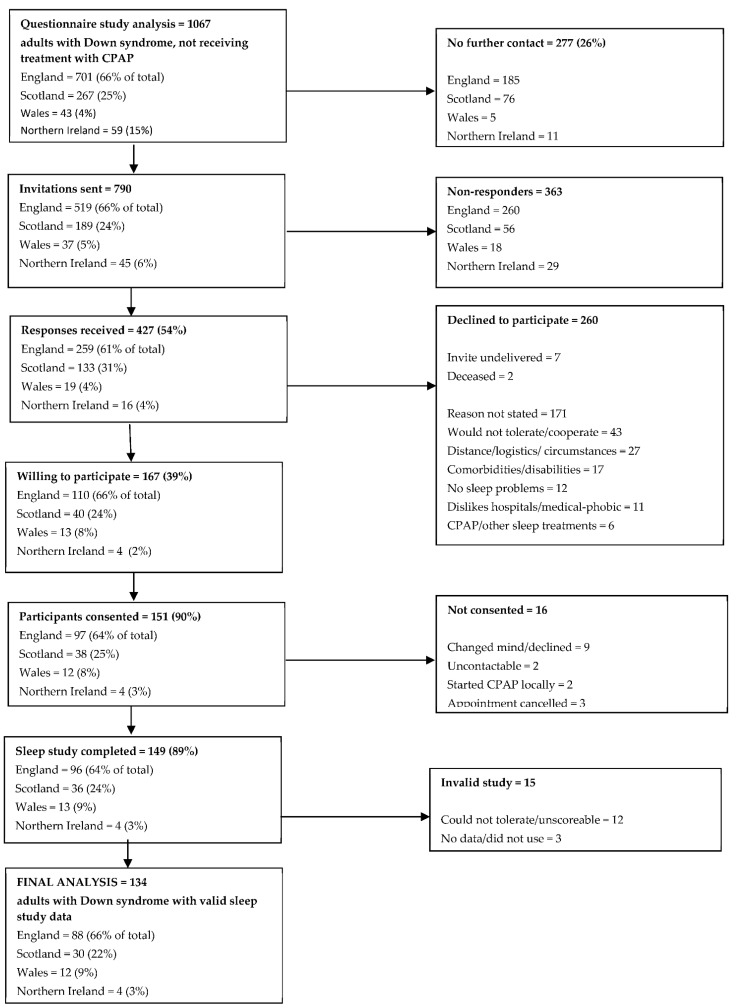
Summary of questionnaire responders entering the objective prevalence study.

A summary of questionnaire responders entering the objective prevalence study is shown in Figure 2. Of the 1067 valid responders not currently receiving CPAP therapy, 277 (26%) did not wish to be contacted further. Of the 790 invitations sent, 427 responses were received (54%), 260 of which declined to participate. Although a reason for declining was not sought, a number of responders annotated their reply slip with a reason; common reasons for declining participation included belief that the individual with Down syndrome would not tolerate the equipment or cope with the study (17%), the logistics of the study or general family circumstances making participation problematic (10%), other co-existing comorbidities or disabilities (7%), and a general dislike of hospitals/medical intervention (4%). Twelve individuals (5%) declined to participate because they did not perceive themselves to have a sleep problem. Six individuals reported that they were on CPAP or other sleep treatments and so were ineligible for further inclusion.

One hundred and sixty-seven individuals were willing to undergo a home sleep study. Of these, 151 (90%) completed informed consent and were formally recruited into the study. Of those who did not consent, nine changed their mind and declined to participate, two could not be contacted to book an appointment and a further two had commenced CPAP through a local clinical service since returning a reply slip. Three individuals cancelled appointments and were not rebooked.

In total, 149 individuals underwent home sleep studies. Of the recorded studies, 15 studies (10%) did not yield valid results, with 12 studies being unscoreable or not tolerated, and 3 studies having no data recorded or not been used and were not repeated. Therefore, the final analysis is based on 134 adults with Down syndrome with valid home sleep study data. 

The total scored recording time averaged 488.4 ± 115.1 min (8.1 ± 2.6 h), similar to the estimated TST at night reported subjectively via questionnaire (8.4 ± 1.2 h), and did not differ significantly between genders. An AHI ≥ 15 or an ODI ≥10/h is considered diagnostic of OSA; 62% met the criteria for AHI and 41% the criteria for ODI. The probable OSAHS algorithms [14] were adapted, to allow direct comparison of the subjective diagnosis of probable OSAHS with the objective diagnosis of OSAHS by substituting snoring ≥ 3 nights/week and witnessed apnoeas with AHI ≥ 15:

1. AHI ≥ 15 plus pESS > 10/24
2. AHI ≥ 15 plus unrefreshing sleep ≥ 3 nights per week.
3. AHI ≥ 15 plus daytime sleepiness ≥ 3 nights per week.

Using these modified algorithms, 42% of participants demonstrated OSAHS on ≥1 definition (versus 56% of the same group who met the criteria for probable OSAHS).

Subjective and objective measures of OSAHS were compared. Sensitivity of the probable OSAHS algorithms (ability of the algorithms to correctly identify individuals with objectively diagnosed OSAHS) was 79.2%, with a specificity (ability of the algorithms to correctly identify individuals who do not have OSAHS) of 58.5%. The positive predictive value (PPV; the likelihood of an individual to have OSAHS in the event of meeting the criteria for probable OSAHS via the algorithms) was 58.5% and the negative predictive value (NPV; the likelihood of an individual to be OSAHS negative in the event of obtaining a negative result on the algorithms) was 79.2%. The likelihood ratio (how much more likely it is that an individual who is positive for probable OSAHS via the algorithms will have an objective diagnosis of OSAHS) was 1.9.

## 4. Discussion 

This is the first large-scale prevalence study of OSAHS in adults with Down syndrome. Based on self-reported symptoms [14], the prevalence of OSAHS was approximately 37%, of whom only 38/404 (9%) had received a prior diagnosis. Using objective methods, a similar prevalence of 42% was observed

This mean prevalence of 40% is modest in comparison with the >80% quoted in previous prevalence studies for the Down syndrome population, though these studies were limited by being extremely small scale [7,8]. The 40% prevalence is substantially higher than the 2–4% prevalence reported in the general population [4]. Clearly, OSAHS is being markedly overlooked in the clinical care of adults with Down syndrome, with only 3% of this cohort already diagnosed with and receiving treatment for OSAHS. This may be related to diagnostic overshadowing, leading to potentially inappropriate treatments for behavioural and emotional disturbances which may result directly from poor sleep but are not being recognised as such. 

### 4.1. Acceptability of Home Sleep Apnoea Testing 

Home sleep apnoea testing with the Embletta^®^ Gold™ cardiorespiratory polygraphy device was well-tolerated by the participants, with a success rate of 90% over two nights’ recording. This is similar to previously published data on the use of home sleep apnoea testing equipment in the general population [18]. Home sleep apnoea testing is commonplace clinically in the UK and Europe, offering logistical as well as financial benefits over inpatient polysomnography [15]. People with Down syndrome may be medical-phobic or find in-patient visits distressing, so adequate testing in the home environment to make a diagnosis of OSAHS as accessible as possible is vital. 

### 4.2. Use of the pESS in Down Syndrome

A discrepancy was noted between self-reporting of daytime sleepiness per se and the percentage of those reporting elevated pESS scores. Although the pESS appears to be a useful measure in this population, overall scores may have been reduced due to the unsuitability of some of the questions. The question regarding sitting down and reading may not be appropriate given the diminished literacy in this population, and may be problematic in those with visual impairment. With regard to the question related to afternoon napping, 69% of responders indicated via the pESS that they were likely to nap in the afternoon should circumstances permit, despite only 28% of respondents reporting daytime napping. This may reflect a lack of opportunity to nap (due to employment, education or other daytime commitments) rather than absence of sleepiness. Modification of the pESS to improve its utility in this specific population may be appropriate.

### 4.3. Self-Reporting of Nocturnal Symptoms

A large percentage of responders did not know whether or not they had apnoeas, which may have resulted in underestimation of the prevalence of this symptom. This may reflect the relative complexity of the description (people are generally more familiar with snoring than “pauses in breathing”), but may also relate to the availability of a second individual to witness the apnoeas; many people with Down syndrome live in supported accommodation without a live-in caregiver or family member and, whereas snoring may be heard outside the bedroom, apnoeas are not. This may also explain the relatively young mean age of respondents, who may still reside at home. However, this is speculative, given that information on living arrangements was not recorded in this study.

All responders with probable OSAHS reported snoring ≥ 3 nights per week, as this variable was common to all three algorithms. Sleep apnoea without snoring is rare in the literature, though one polysomnographic study in adults with Down syndrome [19] noted snoring in only 7 of 12 participants with studies diagnostic of sleep apnoea. The algorithms used in the current study may be under-estimating the prevalence of OSAHS by excluding those who snored < 3 nights per week.

### 4.4. Behavioural and Emotional Disturbances

Generally, scores on the subscales of the DBC-A were low. A floor effect was noted on the Anxiety/Antisocial subscale, with mean scores of 0/14 in both males and females, regardless of sleep symptoms. There may be an element of selection bias, with families of individuals with more severe behavioural and emotional problems less likely to respond. However, probable OSAHS was associated with significant increases in raw and mean scores as well as the breadth and intensity of problem behaviours across all three subscales, supporting the hypothesis of OSAHS impacting negatively on behaviour and emotion in adults with Down syndrome. This may lead to inappropriate prescribing of psychotropic drugs. Given their side effects, and the difficulties that people with Down syndrome may have in reporting side effects due to cognitive and communication impairments, this may cause further unnecessary suffering.

Cognitive and behavioural deficits in adults and children with untreated OSAHS are well documented in the general adult population, as is reversal of these deficits with treatment [4]. Since adults with Down syndrome already exhibit cognitive impairment, untreated sleep-disordered breathing may present a “double-hit” on cognition in these individuals. It is possible that untreated sleep-disordered breathing may contribute to the acceleration of the cognitive decline seen in early onset dementia, which is common in adults with Down syndrome [3]; a recent review by Fernandez and Edgin [7] suggests that sleep disruption might lead to both earlier onset of dementia and more rapid deterioration. However, to date, no published studies have investigated the effect of sleep-disordered breathing on cognitive function in adults with Down syndrome, nor the effect of CPAP therapy in this group. A recent paper by Cody et al. [20] has suggested that disrupted sleep in adults with Down syndrome was more likely to be associated with impaired cognitive functioning and higher striatal beta amyloid deposition. 

### 4.5. Adenotonsillectomy

Adenotonsillectomy is the first-line treatment for OSAHS in the majority of typically developing children, curative in 75–100%, although previous studies report less favourable results in children with Down syndrome [21]. In our study, previous surgery did not result in a lower rate of excessive daytime sleepiness, and those who had previously had adenoids and/or tonsils removed were more likely to exhibit witnessed apnoeas and trended towards reporting more Disruptive and Depressive behaviour. Whilst surgery may result in a partial or initial improvement, results are not sustained into adulthood in the Down syndrome population. This is supported by a previous study in a population of adolescents and young adults (age 14–30 years) with Down syndrome [22]. 

### 4.6. Limitations of the Study

Inherent non-responder bias in questionnaire studies is well-documented, and difficult to avoid [23]; however, we believe that the questionnaire was designed in such a way as to minimise this. An element of selection bias may be evident, with those individuals and families with concerns about sleep more likely to respond.

There was some regional variation in method. An England-based charity which sent out the majority (3895; 74%) of the questionnaires in England, Wales and Northern Ireland declined to send out a second questionnaire to individuals who did not respond initially. A repeat mailout was conducted by all other services involved in the study. This may have contributed to the reduced response rate in these countries. 

Although the questionnaire was designed to be completed by the individual with Down syndrome, it is likely that a large proportion of the questionnaires were completed by a proxy on the participant’s behalf. A “proxy effect” has been reported in the literature [24]. However, since this more often results in under-reporting of characteristics, it is likely that our estimates of prevalence, sleepiness and behaviour are conservative. The approach we utilized in determining a positive or negative diagnosis of OSAHS appeared to be reasonable, but diagnosis by questionnaire and self-report is always fraught with bias, and objective measurement of sleep disordered breathing is always preferable. In the future, better questionnaire methods may be devised that are more specific to the Down syndrome group. 

The majority of participants were identified through patient support groups, and so may not be representative of the Down syndrome population as a whole. Older adults may be under-represented, possibly due to inability to complete the questionnaire on account of comorbidities such as dementia, or absence of a living relative or family member to assist with completion.

One final comment regarding the validity of our results pertains to non-responders. At the time of our study, we did not have the facility to sample non-responders with respect to symptoms of sleep disordered breathing in order to assess whether the prevalence was equally high—a bias which should be controlled for in future studies. 

Information on ethnicity was not collected, and our prevalence data may not be transferable to other countries with divergent ethnicity. However, work comparing the prevalence of OSAHS in adults with Down syndrome in Scotland with that in Japan showed similar prevalence of symptoms, despite the ethnic and anthropometric differences between these two populations [25]. 

This study focussed only on symptoms of OSAHS. However, several symptoms such as excessive daytime somnolence, unrefreshing sleep and frequent night wakening are common to other sleep disorders, which cannot be ruled out as co-morbid or alternative causes for these symptoms. Questionnaire assessment of any disorder, including OSAHS, generally results in a lower specificity than sensitivity and we advocate testing all patients where there is any suspicion of sleep-disordered breathing to ensure it is not missed. 

Building on this work, objective sleep study data could further quantify the severity of sleep-disordered breathing in adults with Down syndrome. The reliability of home sleep apnoea testing is now validated for the diagnosis of sleep disordered breathing in adults in comparison to polysomnography [15]. However, it should be noted that there is, in general, a difference of about 20% between home testing and in-lab PSG with respect to the apnoea–hypopnoea index [26]. Although patients with OSAHS and Down syndrome should be offered treatment with continuous positive airway pressure therapy, a strong evidence base for this was lacking at the time our study was conceived of and commenced (2010–2011). 

## 5. Conclusions

In conclusion, this first large-scale study of OSAHS prevalence in adults with Down syndrome shows an estimate of 37%—nearly 9 times higher than in the general adult population. Unfortunately, neither assessment nor treatment of OSAHS in adults with Down syndrome appears to be common clinical practice, as evidenced by only 7% of the study population having a prior diagnosis of OSA and 3.4% receiving CPAP treatment, despite the potential benefits for improved cognitive function, health and wellbeing. This study strengthens the evidence for guidelines for the monitoring of OSAHS in Down syndrome adults and the establishment of specialised clinics for adults with Down syndrome and other forms of intellectual disabilities. We argue for improved access to these services and measures to improve awareness of this disorder amongst people with Down syndrome, their families and all professionals involved in their care.

## Figures and Tables

**Table 1:** Anthropometric characteristics of all valid questionnaire responders, with responders on CPAP therapy excluded. Chi-square test used for parametric categorical variables, *t*-test for continuous categorical variables and Mann–Whitney U test for non-parametric variables. Values presented as mean ± SD or *n* % unless otherwise stated.

Characteristics	Total Responses	All Responders		Male		Female		*p* **
		*n* = 1067		*n* = 585 (54.8%) *		*n* = 480 (45.0%) *		
Age (years)	1062	28 ± 9		28 ± 9		28 ± 9		0.99
Body Mass Index (kg/m^2^) ***	911	29.0 ± 6.8		28.2 ± 6.6		30.0 ± 6.8		<0.0001
Underweight (<18.5 kg/m^2^)	744	6	0.8%	3	0.7%	3	0.9%	0.001
Normal weight (18.5–24.99 kg/m^2^)		187	25.1%	124	30.1%	63	19.0%	
Pre-obesity (25.0–29.99 kg/m^2^)		255	34.3%	147	35.7%	107	32.3%	
Obesity class I (30.0–34.99 kg/m^2^)		154	20.7%	74	18.0%	80	24.2%	
Obesity class II (35.0–39.99 kg/m^2^)		91	12.2%	43	10.4%	48	14.5%	
Obesity class III (≥40.00 kg/m^2^)		51	6.9%	21	5.1%	30	9.1%	
Collar size (cm)	579	40.4 ± 4.3		41.3 ± 3.8		38.2 ± 4.5		<0.0001
Smoking status:								
Smoker	1017	1	0.1%	1	0.2%	0	0.0%	0.54
Ex-smoker		5	0.5%	2	0.4%	3	0.6%	
Non-smoker		1011	99.4%	548	99.5%	462	99.4%	
Any medication	1067	728	68.2%	369	63.1%	359	74.8%	<0.0001
Comorbidities:								
Asthma	1067	135	12.7%	75	12.8%	60	12.5%	0.93
Adenoid and/or tonsil surgery	1067	249	23.3%	136	23.2%	113	23.5%	0.94
Stroke	1067	16	1.5%	7	1.2%	9	1.9%	0.45
Broken nose	1067	8	0.7%	6	1.0%	2	0.4%	0.31
Diabetes	1067	29	2.7%	13	2.2%	16	3.3%	0.34
Heart problems	1067	37.2	3.5%	182	31.1%	215	44.8%	<0.0001
Hay fever	1067	193	18.1%	104	17.8%	89	18.5%	0.75
Thyroid problems	1067	379	35.5%	168	28.7%	210	43.8%	<0.0001
Epilepsy	1067	62	5.8%	31	5.3%	31	6.5%	0.43
Liver problems	1067	17	1.6%	13	2.2%	4	0.8%	0.09
Hypertension	1067	19	1.8%	9	1.5%	10	2.1%	0.64
Nasal surgery	1067	15	1.4%	8	1.4%	7	1.5%	1.00
Kidney problems	1067	23	2.2%	11	1.9%	12	2.5%	0.53
Gluten intolerance	1067	61	5.7%	26	4.4%	35	7.3%	0.06

* Gender of 2 responders unknown; ** Difference between males and females; *** WHO BMI category calculated for participants aged ≥20 years only.

**Table 2:** Sleep and behaviour characteristics of responders meeting criteria for probable OSA on ≥1 algorithm, with responders on CPAP therapy excluded. Chi-square test used for parametric categorical variables, *t*-test for continuous categorical variables and Mann–Whitney U test for non-parametric variables. Values presented as mean ± SD, median (IQR) or *n* % unless otherwise stated.

Characteristics	Total Responses	Probable OSA		OSA Not Suspected		*p*
		*n* = 366 (34.3%) *		*n* = 673 (63.1%) *		
Prior diagnosis of obstructive sleep apnoea (OSA)	1039	31	8.5%	13	1.9%	<0.0001
DBC-A Disruptive subscale (scale range 0–34)	1023	7 (3–12)		4 (1–8)		<0.0001
Mean item score (possible score 0–2)	1023	0.42 (0.18–0.71)		0.24 (0.06–0.50)		<0.0001
Proportion of items checked (possible score 0–1)	1023	0.41 (0.18–0.59)		0.24 (0.06–0.41)		<0.0001
Intensity index (possible score 0–1)	895	0.10 (0.00–0.33)		0.00 (0.00–0.22)		<0.0001
DBC-A Anxiety/Antisocial subscale (scale range −2–14)	1021	0 (−1–2)		0 (0–1)		0.047
Mean item score (possible score 0–2)	1021	0.22 (0.11–0.44)		0.11 (0.00–0.33)		<0.0001
Proportion of items checked (possible score 0–1)	1021	0.22 (0.11–0.33)		0.11 (0.00–0.22)		<0.0001
Intensity index (possible score 0–1)	766	0.25 (0.00–0.50)		0.00 (0.00–0.50)		0.001
DBC-A Depressive subscale (scale range 0–18)	1024	3 (1–6)		1 (0–4)		<0.0001
Mean item score (possible score 0–2)	1024	0.33 (0.11–0.67)		0.11 (0.00–0.44)		<0.0001
Proportion of items checked (possible score 0–1)	1024	0.33 (0.11–0.56)		0.11 (0.00–0.33)		<0.0001
Intensity index (possible score 0–1)	718	0.00 (0.00–0.39)		0.00 (0.00–0.25)		0.002
Pictorial Epworth Sleepiness Scale (pESS)	933	9 ± 6		6 ± 5		<0.0001
Pictorial Epworth Sleepiness Scale score >10	933	126	38.8%	89	14.6%	<0.0001
Estimated total sleep time (TST) in 24 h (h)	545	9.2 ± 1.5		9.0 ± 1.3		0.10
Estimated TST during night (h)	988	8.5 ± 1.3		8.7 ± 1.2		0.02
Estimated TST during daytime (h)	834	0.0 (0.0–0.1)		0.0 (0.0–0.0)		<0.0001
Naps in daytime	812	111	40.8%	123	22.8%	<0.0001
Snoring—ever (≥1 night/week)	1038	366	100.0%	450	67.0%	-
Never		0	0.0%	149	22.2%	<0.0001
Rarely/sometimes (1–2 night/week)		0	0.0%	396	58.9%	
Often/frequent (≥3 nights/week)		366	100.0%	54	8.0%	
Don’t know		0	0.0%	73	10.9%	-
Witnessed apnoeas—ever (≥1 night/week)	1017	206	56.9%	103	15.7%	-
Never		20	5.5%	339	51.8%	<0.0001
Rarely/sometimes (1–2 night/week)		82	22.7%	78	11.9%	
Often/frequent (≥3 nights/week)		124	34.3%	25	3.8%	
Don’t know		136	37.6%	213	32.5%	-
Nocturnal choking episodes—ever (≥1 night/week)	1021	160	44.9%	117	17.6%	-
Never		145	40.7%	495	74.4%	<0.0001
Rarely/sometimes (1–2 night/week)		111	31.2%	105	15.8%	
Often/frequent (≥3 nights/week)		49	13.8%	12	1.8%	
Don’t know		51	14.3%	53	8.0%	-
Frequent night awakenings—ever (≥1 night/week)	1021	292	81.1%	434	65.7%	-
Never		36	10.0%	195	29.5%	<0.0001
Rarely/sometimes (1–2 night/week)		158	43.9%	316	47.8%	
Often/frequent (≥3 nights/week)		134	37.2%	118	17.9%	
Don’t know		32	8.9%	32	4.8%	-
Unrefreshing sleep—ever (≥1 night/week)	1027	313	86.2%	431	64.9%	-
Never		18	5.0%	188	28.3%	<0.0001
Rarely/sometimes (1–2 night/week)		118	32.5%	299	45.0%	
Often/frequent (≥3 nights/week)		195	53.7%	132	19.9%	
Don’t know		32	8.8%	45	6.8%	-
Daytime sleepiness—ever (≥1 night/week)	1029	322	88.7%	448	67.3%	-
Never		36	9.9%	205	30.8%	<0.0001
Rarely/sometimes (1–2 night/week)		150	41.3%	335	50.3%	
Often/frequent (≥3 nights/week)		172	47.4%	113	17.0%	
Don’t know		5	1.4%	13	2.0%	-

* OSA probability could not be calculated for 28 responders (2.6%).

**Table 3:** Anthropometric characteristics of responders meeting criteria for probable OSA on ≥1 algorithm, with responders on CPAP therapy excluded. Chi-square test used for parametric categorical variables, *t*-test for continuous categorical variables and Mann–Whitney U test for non-parametric variables. Values presented as mean ± SD or *n* % unless otherwise stated.

Characteristics	Total Responses	Probable OSA		OSA Not Suspected		*p*
		*n* = 366 (34.3%) *		*n* = 673 (63.1%) *		
Age (years)	1004	26 ± 8		29 ± 10		<0.0001
Gender (males:females)	1037	206:159		363:309		0.47
Collar size (cm)	565	41 ± 4.6		40.1 ± 4.0		0.02
Body Mass Index (kg/m^2^) **	884	30.0 ± 1.3		27.4 ± 1.2		<0.0001
Underweight (<18.5 kg/m^2^)	721	0	0.0%	6	1.3%	<0.0001
Normal weight (18.5–24.99 kg/m^2^)		42	17.3%	140	29.3%	
Pre-obesity (25.0–29.99 kg/m^2^)		81	33.3%	168	35.1%	
Obesity class I (30.0–34.99 kg/m^2^)		48	19.8%	100	20.9%	
Obesity class II (35.0–39.99 kg/m^2^)		44	18.1%	42	8.8%	
Obesity class III (≥40.00 kg/m^2^)		28	11.5%	22	4.6%	
Any medication	1039	273	74.6%	473	70.3%	0.001
Benzodiazepines/Z-drugs	1039	7	1.9%	6	0.9%	0.24
Opiates	1039	6	1.6%	6	0.9%	0.36
Antidepressants	1039	21	5.7%	33	4.9%	0.56
Antiepileptics	1039	24	6.6%	19	2.8%	0.005
Antihistamines	1039	35	9.6%	36	5.3%	0.01
Contraceptives	1039	29	7.9%	33	4.9%	0.06
Melatonin	1039	7	1.9%	9	1.3%	0.60
Oxygen	1039	4	1.1%	6	0.9%	0.75
Comorbidities:						
Asthma	1039	64	17.5%	68	10.1%	0.001
Stroke	1039	1	0.3%	14	2.1%	0.03
Broken nose	1039	1	0.3%	6	0.9%	0.43
Diabetes	1039	7	1.9%	20	3.0%	0.42
Heart problems	1039	152	41.5%	231	34.3%	0.02
Hay fever	1039	79	21.6%	111	16.5%	0.04
Thyroid problems	1039	130	35.5%	242	36.0%	0.95
Epilepsy	1039	35	9.6%	25	3.7%	<0.0001
Liver	1039	2	0.5%	13	1.9%	0.10
Hypertension	1039	10	2.7%	8	1.2%	0.08
Nasal surgery	1039	6	1.6%	8	1.2%	0.58
Kidney problems	1039	13	3.6%	9	1.3%	0.02
Gluten intolerance	1039	20	5.5%	39	5.8%	0.89
Any adenoid and/or tonsil surgery	1039	105	28.7%	135	20.1%	0.002

* OSA probability could not be calculated for 28 responders (2.6%); ** WHO BMI category calculated for participants aged ≥20 years only.

**Table 4:** Self-reported sleep and behaviour characteristics of valid questionnaire responders, with responders on CPAP therapy excluded. Chi-square test used for parametric categorical variables, *t*-test for continuous categorical variables and Mann–Whitney U test for non-parametric variables. Values presented as mean ± SD, median (IQR) or *n* % unless otherwise stated.

Sleep and Behaviour Characteristics	Total Responses	All Responders		Male		Female		*p* **
		*n* = 1067		*n* = 585 (54.8%) *		*n* = 480 (45.0%) *		
Developmental Behaviour Checklist for Adults (DBC-A):								
Disruptive behaviour subscale (scale range 0–34)	1050	5 (2 to 10)		4 (2 to 9)		6 (2 to 11)		0.003
Mean item score (possible score 0–2)	1050	0.29 (0.12 to 0.59)		0.27 (0.12 to 0.55)		0.35 (0.16 to 0.65)		0.002
Proportion of items checked (possible score 0–1)	1050	0.29 (0.12 to 0.53)		0.24 (0.12 to 0.47)		0.29 (0.12 to 0.53)		0.001
Intensity index (possible score 0–1)	920	0.00 (0.00 to 0.27)		0.00 (0.00 to 0.29)		0.00 (0.00 to 0.27)		0.55
Anxiety/Antisocial subscale (scale range −2–14)	1035	0 (0 to 1)		0 (0 to 1)		0 (−1 to 1)		0.96
Mean item score (possible score 0–2)	1035	0.22 (0.00 to 0.33)		0.22 (0.00 to 0.33)		0.22 (0.11 to 0.33)		0.66
Proportion of items checked (possible score 0–1)	1035	0.11 (0.00 to 0.33)		0.11 (0.00 to 0.33)		0.11 (0.11 to 0.33)		0.64
Intensity index (possible score 0–1)	783	0.00 (0.00 to 0.50)		0.00 (0.00 to 0.50)		0.00 (0.00 to 0.50)		0.91
Depressive subscale (scale range 0–18)	1050	2 (0 to 5)		2 (0 to 5)		2 (0 to 5)		0.17
Mean item score (possible score 0–2)	1050	0.22 (0.00 to 0.56)		0.22 (0.00 to 0.56)		0.22 (0.00 to 0.56)		0.04
Proportion of items checked (possible score 0–1)	1050	0.22 (0.00 to 0.44)		0.22 (0.00 to 0.44)		0.22 (0.00 to 0.44)		0.03
Intensity index (possible score 0–1)	735	0.00 (0.00 to 0.33)		0.00 (0.00 to 0.33)		0.00 (0.00 to 0.33)		0.99
Pictorial Epworth Sleepiness Scale (pESS)	954	7 ± 5		7 ± 6		7 ± 5		0.02
Pictorial Epworth Sleepiness Scale score >10	954	215	22.5%	124	23.8%	90	20.9%	0.31
Estimated total sleep time (TST) in 24 h (h)	559	9.1 ± 1.3		8.9 ± 1.4		9.2 ± 1.3		0.004
Estimated TST during night (h)	1011	8.6 ± 1.2		8.5 ± 1.3		8.8 ± 1.2		<0.0001
Estimated TST during daytime (h)	834	0 (0 to 0.5)		0 (0 to 0.5)		0 (0 to 0.5)		0.96
Naps in daytime	834	235	28.2%	125	27.9%	109	28.3%	0.94
Snoring—ever (≥1 night/week)	1052	830	78.9%	462	80.1%	366	77.4%	*-*
Never		149	14.2%	75	13.0%	74	15.6%	0.41
Rarely/sometimes (1–2 night/week)		396	37.6%	224	38.8%	171	36.2%	
Often/frequent (≥3 nights/week)		434	41.3%	238	41.2%	195	41.2%	
Don’t know		73	6.9%	40	6.9%	33	7.0%	*-*
Witnessed apnoeas—ever (≥1 night/week)	1029	309	30.0%	175	30.8%	133	29.0%	*-*
Never		370	36.0%	200	35.2%	169	36.8%	0.19
Rarely/sometimes (1–2 night/week)		160	15.5%	83	14.6%	76	16.6%	
Often/frequent (≥3 nights/week)		149	14.5%	92	16.2%	57	12.4%	
Don’t know		350	34.0%	193	34.0%	157	34.2%	*-*
Nocturnal choking episodes—ever (≥1 night/week)	1042	282	27.1%	142	24.9%	140	29.9%	-
Never		656	63.0%	362	63.4%	292	62.3%	0.37
Rarely/sometimes (1–2 night/week)		221	21.2%	111	19.4%	110	23.5%	
Often/frequent (≥3 nights/week)		61	5.9%	31	5.4%	30	6.4%	
Don’t know		104	10.0%	67	11.7%	37	7.9%	-
Frequent night awakenings—ever (≥1 night/week)	1044	743	71.2%	393	68.7%	349	74.3%	-
Never		237	22.7%	235	41.1%	101	21.5%	0.36
Rarely/sometimes (1–2 night/week)		484	46.4%	262	45.8%	222	47.2%	
Often/frequent (≥3 nights/week)		259	24.8%	131	22.9%	127	27.0%	
Don’t know		64	6.1%	44	7.7%	20	4.3%	-
Unrefreshing sleep—ever (≥1 night/week)	1047	760	72.6%	413	72.3%	470	73.0%	-
Never		210	20.1%	114	20.0%	95	20.0%	0.90
Rarely/sometimes (1–2 night/week)		428	40.9%	236	41.3%	192	40.5%	
Often/frequent (≥3 nights/week)		332	31.7%	177	31.0%	154	32.5%	
Don’t know		77	7.4%	44	7.7%	33	7.0%	-
Daytime sleepiness—ever (≥1 night/week)	1050	787	75.0%	443	93.1%	342	72.5%	-
Never		244	23.2%	121	25.4%	123	26.1%	0.11
Rarely/sometimes (1–2 night/week)		500	47.6%	276	58.0%	224	47.5%	
Often/frequent (≥3 nights/week)		287	27.3%	167	35.1%	118	25.0%	
Don’t know		19	1.8%	12	2.5%	7	1.5%	-
Obstructive sleep apnoea (OSA) status:								
Prior diagnosis of OSA	1067	44	4.1%	29	5.0%	15	3.1%	0.16
Probable OSA using definition 1	1038	350	33.7%	199	34.9%	150	32.2%	0.39
Probable OSA using definition 2	1042	356	34.2%	200	35.1%	155	33.0%	0.51
Probable OSA using definition 3	1038	352	33.9%	200	35.1%	151	32.4%	0.39
Probable OSA on ≥1 definition	1039	366	35.2%	206	36.2%	159	34.0%	0.47

* Gender of 2 responders unknown; ** Difference between males and females.

**Table 5:** Determinants of sleepiness and probable obstructive sleep apnoea/hypopnoea syndrome as assessed by binary logistic regression (OR) * or generalised linear modelling (β) ** as appropriate for categorical and continuous variables, respectively. DBC-A Disruptive subscale analysed separately for males and females due to significant difference in scores at baseline.

Variable	Total Included	Determinants Remaining in Model	Estimate	95% CI Lower	95% CI Upper	*p*
Pictorial Epworth Sleepiness Scale **		Age	0.0	0.0	0.1	0.07
		BMI	0.1	0.0	0.2	0.004
		Hay fever	1.3	0.2	2.4	0.02
		Category G medication	2.2	0.5	3.9	0.01
		Category N medication	2.0	0.8	3.1	0.001
		Witnessed apnoeas—rarely/sometimes (1–2 nights/week)	1.8	0.8	2.8	0.001
		Witnessed apnoeas—often/frequently (≥3 nights/week)	5.5	4.4	6.6	<0.0001
Excessive daytime sleepiness (pESS > 10) *		Age	1.0	1.0	1.1	0.14
		BMI	1.0	1.0	1.1	0.08
		Category N medication	0.4	0.2	0.7	0.001
		Witnessed apnoeas—rarely/sometimes (1–2 nights/week)	0.1	0.1	0.2	<0.0001
		Witnessed apnoeas—often/frequently (≥3 nights/week)	0.3	0.2	0.6	<0.0001
Probable obstructive sleep apnoea/hypopnoea syndrome **		Age	1.0	0.9	1.0	<0.0001
		BMI	1.1	1.1	1.1	<0.0001
		Epilepsy	0.2	0.1	0.5	<0.0001
		Category R medication	0.6	0.4	0.9	0.01

**Table 6:** Determinants of behavioural and emotional disturbances as assessed by generalised linear modelling (β) for continuous variables. DBC-A Disruptive subscale analysed separately for males and females due to significant difference in scores at baseline.

Variable	Total Included	Determinants Remaining in Model	Estimate (β)	95% CI Lower	95% CI Upper	*p*
DBC-A Disruptive—Male		Category N medication	3.0	1.2	4.8	0.001
		Snoring—rarely/sometimes (1–2 nights/week)	−1.8	−3.4	−0.1	0.04
		Snoring—often/frequently (≥3 nights/week)	−1.4	−3.3	0.6	0.16
		Witnessed apnoeas—rarely/sometimes (1–2 nights/week)	2.0	0.3	3.7	0.02
		Witnessed apnoeas—often/frequently (≥3 nights/week)	1.5	−0.5	3.5	0.14
		Nocturnal choking—rarely/sometimes (1–2 nights/week)	1.4	−2.1	3.0	0.09
		Nocturnal choking—often/frequently (≥3 nights/week)	−0.4	−3.2	2.4	0.77
		Frequent awakenings—rarely/sometimes (1–2 nights/week)	1.8	0.3	3.3	0.02
		Frequent awakenings—often/frequently (≥3 nights/week)	3.7	1.7	5.7	<0.0001
		Unrefreshing sleep—rarely/sometimes (1–2 nights/week)	−0.1	−1.7	1.4	0.85
		Unrefreshing sleep—often/frequently (≥3 nights/week)	2.1	−0.2	4.3	0.07
DBC-A Disruptive—Female		Category N medication	4.1	2.6	5.6	<0.0001
		Snoring—rarely/sometimes (1–2 nights/week)	0.6	−1.0	2.2	0.47
		Snoring—often/frequently (≥3 nights/week)	0.5	−1.2	2.3	0.54
		Witnessed apnoeas—rarely/sometimes (1–2 nights/week)	−1.6	−3.1	−0.1	0.04
		Witnessed apnoeas—often/frequently (≥3 nights/week)	−0.8	−3.2	1.5	0.48
		Nocturnal choking—rarely/sometimes (1–2 nights/week)	2.6	1.1	4.1	0.00
		Nocturnal choking—often/frequently (≥3 nights/week)	3.9	1.0	6.7	0.01
		Frequent awakenings—rarely/sometimes (1–2 nights/week)	1.4	−0.1	2.9	0.08
		Frequent awakenings—often/frequently (≥3 nights/week)	−0.2	−2.1	1.7	0.85
		Unrefreshing sleep—rarely/sometimes (1–2 nights/week)	1.5	0.0	3.0	0.06
		Unrefreshing sleep—often/frequently (≥3 nights/week)	6.2	4.3	8.1	<0.0001
DBC-A Anxiety/Antisocial		Category R medication	0.3	0.0	0.6	0.09
		Category N medication	0.1	−0.2	0.4	0.56
		Snoring—rarely/sometimes (1–2 nights/week)	0.0	−0.4	0.3	0.82
		Snoring—often/frequently (≥3 nights/week)	−0.3	−0.7	0.1	0.13
		Witnessed apnoeas—rarely/sometimes (1–2 nights/week)	0.2	−0.2	0.5	0.32
		Witnessed apnoeas—often/frequently (≥3 nights/week)	−0.1	−0.5	0.3	0.67
		Nocturnal choking—rarely/sometimes (1–2 nights/week)	0.3	0.0	0.7	0.03
		Nocturnal choking—often/frequently (≥3 nights/week)	0.5	−0.1	1.1	0.11
		Frequent awakenings—rarely/sometimes (1–2 nights/week)	0.1	−0.2	0.4	0.55
		Frequent awakenings—often/frequently (≥3 nights/week)	0.0	−0.4	0.4	0.87
		Unrefreshing sleep—rarely/sometimes (1–2 nights/week)	0.0	−0.3	0.3	1.00
		Unrefreshing sleep—often/frequently (≥3 nights/week)	0.5	0.1	1.0	0.03
		Daytime sleepiness—rarely/sometimes (1–2 nights/week)	0.2	−0.1	0.5	0.20
		Daytime sleepiness—often/frequently (≥3 nights/week)	0.5	0.1	0.9	0.01
DBC-A Depressive		Category R medication	0.0	−0.7	0.7	1.00
		Category N medication	2.3	1.6	3.0	<0.0001
		Snoring—rarely/sometimes (1–2 nights/week)	−0.2	−0.9	0.5	0.50
		Snoring—often/frequently (≥3 nights/week)	−0.2	−1.0	0.5	0.55
		Witnessed apnoeas—rarely/sometimes (1–2 nights/week)	0.4	−0.3	1.1	0.22
		Witnessed apnoeas—often/frequently (≥3 nights/week)	0.9	0.0	1.8	0.06
		Nocturnal choking—rarely/sometimes (1–2 nights/week)	0.5	−0.2	1.1	0.16
		Nocturnal choking—often/frequently (≥3 nights/week)	0.5	−0.7	1.6	0.46
		Frequent awakenings—rarely/sometimes (1–2 nights/week)	0.5	−0.2	1.1	0.15
		Frequent awakenings—often/frequently (≥3 nights/week)	0.3	−0.6	1.1	0.52
		Unrefreshing sleep—rarely/sometimes (1–2 nights/week)	0.5	−0.2	1.1	0.19
		Unrefreshing sleep—often/frequently (≥3 nights/week)	2.3	1.4	3.3	<0.0001
		Daytime sleepiness—rarely/sometimes (1–2 nights/week)	−0.1	−0.7	0.5	0.67
		Daytime sleepiness—often/frequently (≥3 nights/week)	0.5	−0.3	1.3	0.23

## Data Availability

The authors are happy to share data utilised in the analyses detailed above on personal request to the first or last author and with the full permission of all authors in this study.
